# Fast Periodic Visual Stimulation indexes preserved semantic memory in healthy ageing

**DOI:** 10.1038/s41598-020-69929-5

**Published:** 2020-08-04

**Authors:** Alex Milton, Alesi Rowland, George Stothart, Phil Clatworthy, Catherine M. Pennington, Nina Kazanina

**Affiliations:** 1 School of Psychological Science, University of Bristol, Bristol, BS8 1TU UK; 2 Department of Psychology, University of Bath, Bath, BA2 7AY UK; 3 Department of Neurology, North Bristol NHS Trust, Bristol, BS10 5NB UK; 4 ReMemBr Group, Institute of Clinical Neurosciences, University of Bristol, Bristol, BS10 5NB UK; 5 Centre for Dementia Prevention, University of Edinburgh, Edinburgh, EH16 4UX UK; 6 International Laboratory of Social Neurobiology, Institute of Cognitive Neuroscience, National Research University Higher School of Economics, Moscow, Russian Federation

**Keywords:** Cognitive ageing, Human behaviour

## Abstract

Behavioural studies investigating the preservation of semantic memory in healthy ageing have reported mixed findings. One suggested reason for this discrepancy is that the processes underpinning lexical access to semantic knowledge may be sensitive to ageing. It is therefore necessary to assess semantic memory utilising tasks that are not explicitly linguistic. In this study, a fast periodic visual stimulation (FPVS) paradigm coupled with EEG was used to assess the ability of younger and older adults to automatically distinguish between images by their semantic category. Participants were presented with a 6 Hz stream of images drawn from one semantic category except every fifth image (occurring at a rate of 1.2 Hz) which was drawn from an alternate semantic category. For both younger and older adults, results demonstrate successful and comparable semantic categorisation. This was detectable at the individual level for 71% and 72% of older and younger adults, respectively. Given the rapid presentation rate and absence of explicit instruction to categorise images, the task is unlikely to utilise linguistic strategies and suggests the maintenance of semantic memory in healthy ageing. Moreover, this study utilised mobile EEG equipment and short presentation times that would be suitable for practical application outside a research setting.

## Introduction

Ageing, even in health, is characterised by decline in several cognitive domains. Processing speed^1^, attention (divided, selective and sustained, for a review see^2^ and memory (working and episodic)^3,4^ have all consistently been demonstrated to deteriorate in healthy ageing. The current study examined age effects on another type of memory, namely semantic memory, which stores general (i.e. non-episodic) declarative knowledge accumulated and categorised throughout life. In particular, semantic memory hosts information about taxonomic and associative relations of objects in the world that largely underlies our understanding of these objects^5^. For example, putting something into the category of a fruit in semantic memory makes it possible to make inferences about its properties (edible, sweet), function (can be eaten) and origin (grows on trees), as well as allowing it to be related to other categories of objects such as other food items. In this way semantic memory is critical for valid functioning of the individual in the world around them.

Semantic memory develops in childhood, and already by the child’s second birthday it reflects both taxonomic (e.g., chicken–dog) and associative (e.g., cat–dog) relations, demonstrated in behavioural^6–10^ and in ERP studies^11,12^. Previous research on healthy ageing has demonstrated that richness of semantic network is largely maintained with age. Much of the ageing research on semantic memory has been based on tasks using linguistic stimuli, reflecting the idea that lexico-semantic organisation (i.e. relations between words such as *apple* and *pear*) mirrors the corresponding conceptual semantic organisation (i.e. relations between the concepts ‘apple’ and ‘pear’). Numerous studies found that healthy older adults showed semantic priming effects at least as large as young adults, suggesting unimpaired organisation of the semantic network (even though processing times increased with age)^13–17^. The claim is strengthened by Bennett and McEvoy’s^18^ finding of similar performance in older and younger adults using a mediated semantic priming paradigm in which, rather than being directly semantically related, the prime and target are connected only through another unpresented word (e.g. the relation between the prime *pasture* and the target *milk* is mediated by the word *cow*). These mediated priming effects indicate that spreading activation in the lexico-semantic network is not affected by age. Similarly, in a semantic word association task in which participants were asked to name the first word that came to mind after being presented with a target word, performance of older and younger adults was equivalent once vocabulary size was taken into account^19^.

Yet, consistent reports of the spared contents and structure of semantic memory co-exist with equally robust demonstrations of age-related performance declines in other semantic tasks. In particular, older adults have difficulties in picture naming tasks or when they are asked to produce a word from a definition^20–22^, and they also experience tip-of-the-tongue phenomena more often than younger adults^23^,for a review see^24^. This apparent discrepancy is attributed to performance factors, particularly to age-weakening of retrieval processes rather than to degradation of the structure or content of the semantic memory system itself. The proposed dissociation between structural semantic knowledge that remains intact in ageing vs. executive retrieval processes that deteriorate with age is supported by several experimental studies. For instance, Mayr and Kliegl^25^ asked participants to generate exemplars either from a single semantic category or from two alternating categories. The manipulation between number of categories made it possible to distinguish between semantic search processes that are present in both conditions vs. additional executive function load present specifically in the case of two alternating categories. Whereas the rate of semantic access did not change with age, age-related decline was found in the non-semantic executive task elements.

The extant research into semantic memory predominantly used a linguistic paradigm. Thus a more direct or different assessment of semantic memory that does not rely on the linguistic system would be useful for multiple reasons. Most relevantly, it makes it possible to evaluate whether age-related changes in lexical tasks can indeed be explained by performance factors related to lexical access rather than semantic memory per se. The current electroencephalography (EEG) study followed an earlier study by Stothart et al.^26^ and assessed semantic memory in healthy young and old participants using Fast Periodic Visual Stimulation (FPVS) (a Steady State Visual Evoked Potential technique, for a review see^27^). In our study, participants passively watched images of objects presented centrally and sequentially at a fixed periodic rate of six images per second (6 Hz). This fast presentation rate ensured that the images could not be named (as they could be if the presentation rate were slower), thus tapping into semantic memory without an accompanying need for accessing words. The order of images was pseudo-randomised so that there were four images representing a natural object such as animals and plants (horse, tree, bird, poppy, etc.) and the fifth image represented a man-made object (house, cup, frying pan, etc.). If participants automatically processed semantic properties of depicted objects, they should show a mismatch response to the oddball category, i.e. 1.2 Hz (as an oddball, man-made image appeared every five images, i.e. every 5 × 166.7 ms = 833.3 ms). In their study with healthy young adults, Stothart et al.^26^ found a significant response at the oddball frequency in the power spectrum, demonstrating automatic semantic categorisation of the visual stimuli. If an oddball response of similar magnitude is found in healthy older adults in our study, this would demonstrate an intact semantic memory network in older adults and age constancy both for the organisation of semantic memory and for automatic access of the memory network. Conversely, if semantic memory processes decline in older adults then we would expect a diminished response compared to the younger cohort.

In addition to the theoretical and experimental value, the automatic assessment of sematic memory in older adults has important practical potential with regards to clinical diagnosis of conditions in which semantic memory is affected such as fronto-temporal dementia. The design features of our studies were deliberately chosen so that they are maximally useful with this perspective in mind. To be useful in a clinical context, the proposed assessment method needs to yield data that are interpretable at the level of individual participants (as opposed to most EEG research in which averaging across subjects is needed to obtain interpretable data) and on the basis of a relatively short recording time. The FPVS paradigm that we employ ticks both of these boxes: in a previous study of semantic discrimination, 19/20 healthy young adults had a statistically significant response of the oddball frequency with only 3 min of EEG recording suggesting that the approach has high signal-to-noise ratio that makes it possible to evaluate individual subject responses^26^. Last but not least, we used affordable, relatively low-resolution mobile EEG headsets that require minimal set-up time and thus have potential of being used widely in a clinical setting, for example for patient screening.

## Results

**Figure 1:**
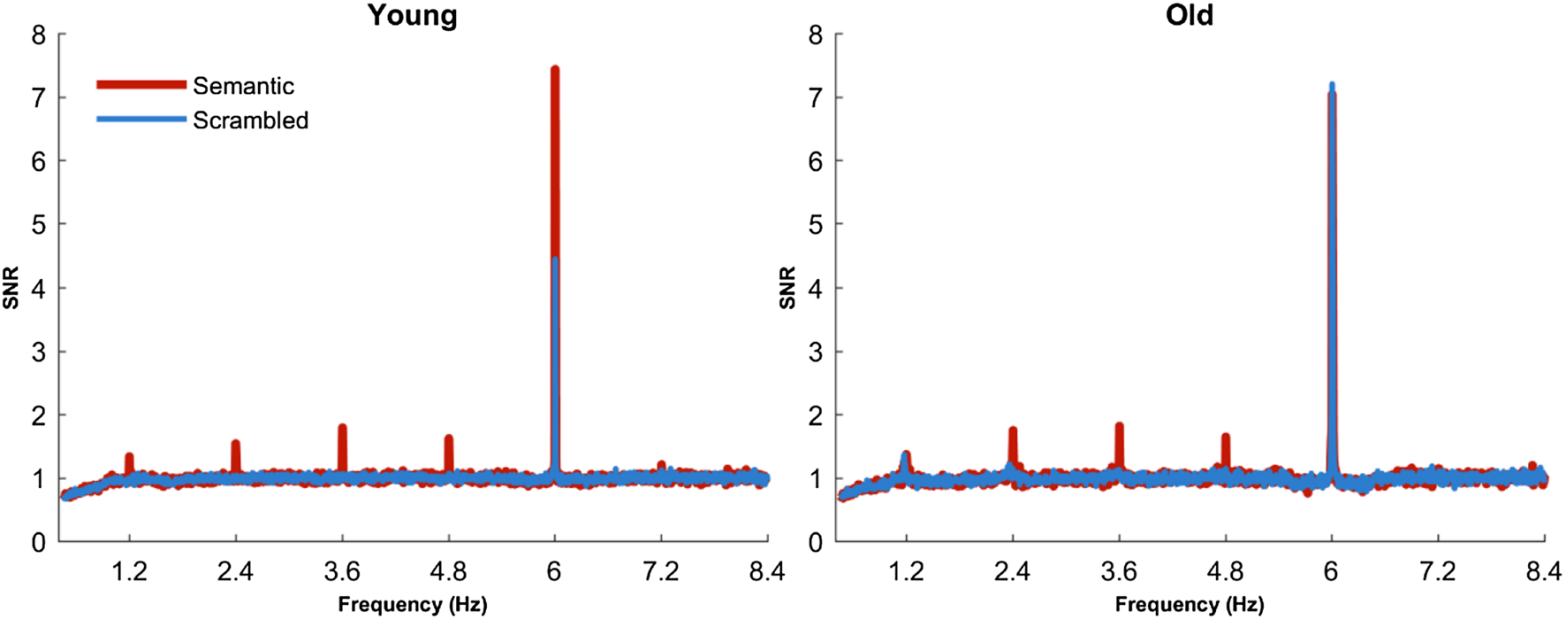
SNR for Semantic and Scrambled conditions for both age cohorts. The SNR is calculated for the global average of all electrodes. The standard presentation rate (*F*) is 6 Hz and the oddball (1.2 Hz) and its harmonics (2.4, 3.6, 4.8…) are displayed up to 8.4 Hz with surrounding frequencies not related to stimulus presentation.

Figure 1 shows the SNRs for the younger and older participants. From inspection of Fig. 1, clear activity is apparent at the base rate frequency (*F*) of 6 Hz as well as the oddball frequency (1.2 Hz) and its harmonics (*f*+) up to 6 Hz. Before turning to *f*+ which is of primary interest to us, it is worth noting that the activity was strongest at the base rate frequency *F*. *Z-*scores for *F* in both the Semantic (global average across participants and electrodes, *M* = 10.99, *SD* = 8.82) and Scrambled condition (*M* = 11.01, *SD* = 4.75) statistically confirm the increased activity at the base rate frequency. For younger adults, Fig. 1 shows a reduction in SNR for *F* in the Scrambled versus Semantic condition whereas this does not seem to be true for older adults. Therefore, an unplanned analysis investigated the effect of age cohort (Young/Old) and condition (Semantic/Scrambled) on activity at *F* using a 2 × 2 mixed ANOVA. As the Shapiro–Wilk test indicated violations of normality, data was log transformed prior to analysis so that normality assumptions were met (Shapiro–Wilk *p* values: Older adults Semantic: *p* = 0.417; Older adults Scrambled: *p* = 0.706; Younger adults Semantic: *p* = 0.841; Younger adults Semantic: *p* = 0.706). Confirming visual inspection, there was a significant Age × Condition interaction (*F*(1,45) = 20.807, *p* < 0.001; *η*_*p*_^2^ = 0.316). Analysis of simple main effects investigated the effect of Condition for each Age group and demonstrated a significant difference in SNR of *F* for younger adults where the Semantic condition was associated with higher SNR values (*M* = 7.446; *SD* = 3.030) than the Scrambled condition (*M* = 4.521; *SD* = 1.677; *p* < 0.001). Whereas there was no condition difference for older adults (*p* = 0.928) between Semantic SNR (*M* = 7.082; *SD* = 2.491) and Scrambled SNR (*M* = 7.273; *SD* = 2.945).

### Main analysis

To investigate the effect of age cohort (Young/Old) and condition (Semantic/Scrambled) on activity at *f*+ we conducted a 2 × 2 mixed ANOVA on the global average amplitude across *f*+. Given the findings concerning the SNR of *F*, we entered the proportional difference in *F* SNR between Semantic and Scrambled conditions as a covariate. This covariate reflects a general reduction in response to scrambled images as opposed to changes in semantic content, e.g. due to the fact that the Scrambled condition always followed the Semantic one. As the Shapiro–Wilk test indicated violations of normality, data was log transformed prior to analysis so that normality assumptions were met (Shapiro–Wilk *p* values: Older adults Semantic: *p* = 0.203; Older adults Scrambled: *p* = 0.579; Younger adults Semantic: *p* = 0.090; Younger adults Semantic: *p* = 0.848).

There was a main effect of condition (*F*(1,44) = 43.403, *p* < 0.001; *η*_*p*_^2^ = 0.497), with a stronger SNR for *f* + in the Semantic condition (*M* = 2.0; 95% CI 1.79–2.04) than Scrambled condition (*M* = 1.48; 95% CI 1.45–1.51). The main effect of Age was not statistically significant (*F*(1,44) = 1.175, *p* = 0.284; *η*_*p*_^2^ = 0.026); nor was the Age × Condition interaction (*F*(1,44) = 0.350, *p* = 0.557; *η*_*p*_^2^ = 0.008). There was no significant effect of proportion change in *F* or any interaction (*p* > 0.1).

As sample sizes were not matched for the two age groups, we randomly sampled a subgroup of younger adults that was equal to the number of older adults (18) and ran a mixed 2 × 2 ANOVA looking at the effect and interaction of Condition and Age on activity at *f*+. As the covariate of proportion change in *F* was not significant in the original analysis, this was not included in the analysis. This procedure was performed 1,000 times on different randomly sampled subgroups of younger adults. We then compared the 1,000 subsampled results (main effects and interaction) to the results of the same 2 × 2 analysis on the original data with an unequal sample size. For the main effect of Age, in 960 of the 1,000 subgroup analyses conducted the main effect was not statistically significant (*p* > 0.05) as reported in our original data analysis. For the main effect of Condition, all 1,000 subgroup analyses were statistically significant with the Semantic condition being associated with a greater response. For the interaction, this was not significant in any of the 1,000 subgroup analyses. In addition, the reported *F* values from the original data analysis were placed in the distribution of *F* values observed in the 1,000 subgroup analyses to see if they fell within the tails of the distribution (< 0.025 or > 0.975) (for histograms of this analysis, see Supplementary Information). This was not the case for the main effect of Age (0.622), Condition (0.861) or the interaction (0.324). Accordingly, we conclude that the unequal sample size in our original analysis did not bias the results.

Although the ANOVA provided no evidence for a difference in SNR response between younger and older adults, the question of whether the data provide more support for equivalent semantic SNR responses versus a reduction of semantic SNR responses in older adults was assessed more directly with a Bayes factor analysis using JASP (JASP Team 2019); JASP (Version 0.11.1). Two models were compared for effect size: the null hypothesis where the semantic SNR response for older and younger adults is equal (effect size of 0); the alternative hypothesis that the semantic SNR response of older adults is smaller than younger adults reflecting reduced semantic processing with age. The prior was located at zero with a Cauchy distribution of 0.707. The Bayes factor provided moderate evidence in favour of the null hypothesis versus the alternative hypothesis (BF_01_ = 4.947; 0.002% error) suggesting that the data better support the idea of equivalent SNR response between younger and older adults in the semantic condition.

### Topographic analysis

Due to a low number of electrodes and relatively sparse placement, we looked at regional effects in *f*+ through a 3-factor mixed ANOVA with Electrode as a 14-level factor, Condition (Semantic/Scrambled) and Age (Old/Young). This analysis included only participants with data for all 14 channels and is therefore a smaller subset than the global average data: 13 older adults and 25 younger adults. As the Shapiro–Wilk test indicated violations of normality, data was log transformed. Transformation reduced violations of normality from 17 to 4 of the 28 data columns in the analysis (Condition × Electrode). For a non-parametric analysis of the Condition by Electrode interaction that supports the ANOVA results reported below, see Supplementary Information.

For the sake of brevity, we report on effects and interactions including the Electrode condition only. Other effects and interactions retained the pattern outlined in the global average analysis outlined above. There was a main effect of Electrode (*F*(13,468) = 14.288, *p* < 0.001; *η*_*p*_^2^ = 0.284) with the SNR of *f*+ showing greatest strength at occipital electrodes (O1/O2). There was an Electrode × Age interaction (*F*(13,468) = 1.909, *p* = 0.027; *η*_*p*_^2^ = 0.050). The first-order effect of Age on *f*+at each electrode was investigated and showed a significant difference (Bonferroni corrected) at electrodes O1 (*F*(1,36) = 4.805, *p* = 0.035; *η*_*p*_^2^ = 0.118), O2 (*F*(1, 36) = 4.193, *p* = 0.048; *η*_*p*_^2^ = 0.104) and P8 (*F*(1,36) = 5.032, *p* = 0.031; *η*_*p*_^2^ = 0.123). In these three electrodes, SNR for *f*+ was higher in older adults than younger adults. For all other electrodes *p* > 0.1.

**Figure 2:**
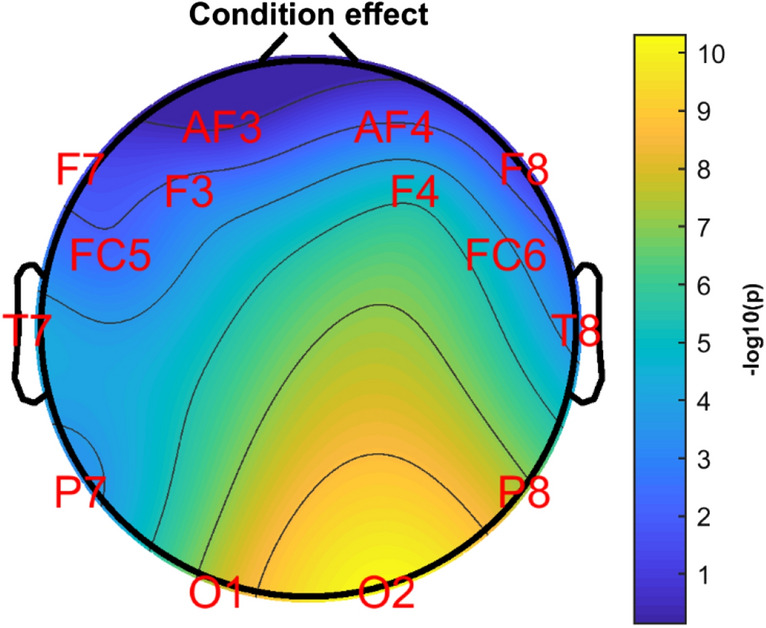
Topographic map showing first-order effects of Condition (Semantic/Scrambled) at each electrode. Colour denotes − log10 of the *p* value for this effect.

The Electrode × Condition interaction was also significant (*F*(13,468) = 7.408, *p* < 0.001; *η*_*p*_^2^ = 0.171). The first-order effect of Condition on *f*+ at each electrode was calculated (Bonferroni corrected) and shown in Fig. 2. The effect of Condition (Semantic/Scrambled) was significant (*ps* < 0.035) at all electrodes except AF3 (*p* = 0.723) and F8 (*p* = 0.218). For all significant electrodes, the semantic condition was associated with a higher *f*+ than the scrambled condition. The Electrode × Condition × Age interaction was not statistically significant (*F*(13,468) = 1.056, *p* = 0.395; *η*_*p*_^2^ = 0.029).

### Individual subject effects

We were interested in examining the strength of individual oddball responses, hence activity at *f*+ in the Semantic and Scrambled conditions in single subjects was explored. This analysis examined the z-scores of *f*+ with a criterion of z = 1.64 (*p* < 0.05, one-tailed) to determine whether amplitude at frequency bins related to the oddball stimulus differed from surrounding bins. This was done at O_2_ as the topographical analysis showed the strongest effect in this electrode. Investigation at this electrode therefore represents an upper-bound of experimental sensitivity. For this analysis, one older adult had data from channel O_2_ excluded due to > 10% noise (see “Methods”), so older adult data is based on 17 participants.

**Figure 3:**
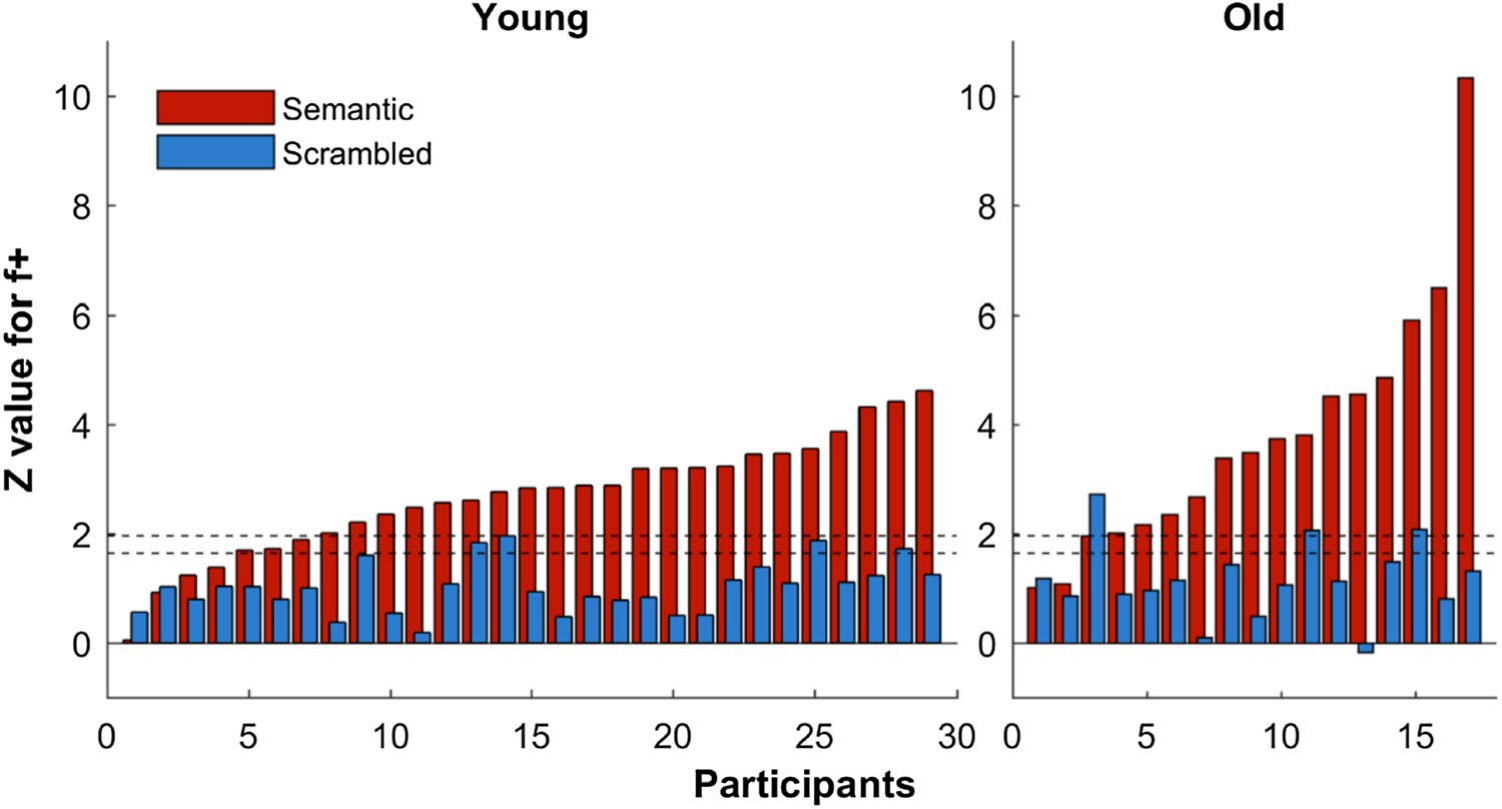
Individual subject *z*-scores for *f*+ (mean of z-scores for all *f*+ frequencies) for Semantic and Scrambled conditions at electrode O2 for Younger and Older adults. Dashed horizontal lines mark z-score threshold for significance at *z* = 1.64 (*p* = 0.05, one-tailed) and *z* = 1.96 (*p* = 0.05, two-tailed).

Z-scores for *f*+ from individual participants are illustrated in Fig. 3 (see Supplementary Information for figures with individual base frequency (*F*) response plotted as well). The key question is: for how many participants can a significant response to the oddball stimulus be observed? On a conservative approach, one would include all participants for whom the Semantic condition—but not the Scrambled condition—reaches the threshold of *z* = 1.64. Using these criteria, 12/17 older adults and 21/ 29 younger adults exhibit effects at the individual level.

## Discussion

We used steady-state visual evoked potentials to assess semantic memory in healthy ageing. Healthy younger and older adults watched sequences of images in which frequent natural objects were regularly interspersed with oddball man-made objects; in the control condition the sequence contained scrambled images of the same objects. A strong response at the base stimulation frequency was found in both conditions and in both age groups, reflecting a basic visual response to images. Critically, a frequency response at the oddball stimulus rate was also present in both age groups and was not affected by age, demonstrating preserved semantic memory organisation and automaticity of semantic categorisation in healthy ageing. This finding represents an important contribution to the existing literature on semantic memory in healthy ageing: unlike most previous studies, a non-verbal task was used and a timescale that made any conscious, strategic or non-strategic access to linguistic information unlikely. Hence previous claims of intact semantic memory and access to it via the verbal route in ageing^13–17^ are complemented with our findings demonstrating intact semantic memory and access to it via the visual ventral stream.

Largely, these group results also hold at the individual level. The frequency response at the base presentation rate was observable in every younger or older participant without exception. More importantly, an oddball response (z >  = 1.64) in the semantic condition could be observed in 25/29 of younger participants and 15/17 older participants using the data from one channel, O2. When comparing the semantic to the scrambled condition, an oddball response (z ≥ 1.64) was found selectively in the semantic condition for 71% and 72% of older and younger adults, respectively. This finding speaks to a remarkable signal-to-noise ratio of the FPVS paradigm that enables identification of an individual-level response even in low-resolution recordings lasting only a few minutes. The simplicity of the criterion used here to assess an individual participant’s oddball response (i.e. the frequency response at the oddball frequency being significantly higher than at an adjacent frequency region at O_2_) adds to the feasibility of using the current design with clinical populations. This technique may be particularly useful in difficult to diagnose dementias such as semantic dementia, aphasia or posterior cortical atrophy. Indeed, the combination of features is useful: a passive task, short duration, robust findings using a low-resolution EEG system, a localisable effect that can be found using only a few EEG channels. A passive task yields further benefits in that it makes the paradigm extendable to populations for which tasks instructions, including young children, patients unable to produce an overt response such as when movement and/or articulatory limitations are involved, as well as to participants for whom the presence of a task causes anxiety or other discomfort.

The current study, together with a previous one^26^, provides support to the claim that semantic categorisation of visual objects is automatic and present even when not required by the task^28,29^, highlighting the fundamental connection between the visual-perceptual system and stored conceptual knowledge. Note, however, that the claim of semantic categorisation as an intrinsic component of early visual processing must be circumscribed. First, semantic categorization may still strictly follow some basic visual detection processing^30^ or even be an outcome of rapid feedback^31^. Second, semantic categorisation refers to an individual’s ability to create equivalence classes which may occur at different levels of specificity (e.g., an eagle classified as a natural object vs. an animal vs. a bird vs. a bird of prey^32^). Rather than being ubiquitously present, the quick automatic classification may be limited to a subset of ‘entry-level’ categories^29^. Admitting limits to automatic semantic categorisation makes it possible to bridge the gap to the conclusions reached in the theoretical^33,34^ and neuro-computational literature^35,36^ that posit categorization as a separate stage that follows early, purely visual, processes.

The response at the oddball frequency obtained using FPVS in this study represents a frequency-domain correlate of the visual mismatch negativity (MMN) ERP component in the time domain^37^, for a review of visual MMN see^38^. In both cases the response is generated by a change-detection mechanism that detects discrepancy between the current stimulus and the short-term memory trace built on the basis of several preceding stimuli^39^. Preservation of visual change detection in ageing has been shown in a MMN study by Stothart et al.^40^, for a review on visual MMN see^41^. Yet, despite the MMN being an automatic response appearing already 120 ms after the stimulus onset, stimulus presentation rate in MMN studies (typically 500–1000 ms per stimulus) is such that each stimulus can be consciously evaluated by the participant. Given the repetitive nature of the paradigm, it cannot be ruled out that participants consciously reflect upon and formulate equivalence classes corresponding to the standard and oddball categories. In contrast, the rate of presentation in the current study is faster and prevents any conscious categorisation, or for this matter, another conscious strategy. The finding of an oddball response using FPVS further strengthen the claim that detection-of-change mechanisms are preserved in ageing. However, we note that our study was powered to detect a medium effect of age. This was based on previous studies that reported a medium effect of age on response accuracy and a large effect of age on response latencies in a word retrieval task^21^. However, we cannot rule out that the true effect size could have been small (the power to observe a small effect with the current sample was 28%) or that the conclusion that there is no effect is a Type 2 error.

It is evident in the results that older adults maintained a similar strength of response to the base rate frequency in both Semantic and Scrambled conditions whereas younger adults showed a reduced response to the base rate frequency *F* in the Scrambled condition. This difference may reflect a decline in inhibitory processing specific to older adults. Stimuli in the scrambled condition had no higher order characteristics, and there was no noticeable periodic variation in the semantic category of the stimuli. For younger adults, *F* in the scrambled condition was significantly reduced compared to the semantic condition, potentially reflecting a reduction in the attentional engagement with the stimuli. Steady state evoked potentials have previously been shown to be moderated by attention^27^, and younger adults may have devoted fewer attentional resources to a constant and meaningless stimuli stream. A characteristic of healthy ageing is a reduction in the ability to inhibit irrelevant information^4,42^ and in a recent study of auditory MMN healthy older adults showed reduced neural inhibition of repeating standard stimuli^43^. We propose that the lack of reduction in *F* in the older adults to the scrambled stimuli may reflect a similar deficit in inhibition, i.e. they did not attentionally downregulate their processing of a meaningless stream of stimuli to the same degree as younger adults. This may also account for the observation that older adults showed oddball-related activity at the first harmonic in the scrambled condition. A reduction in inhibition of this stimulus stream may have led to detection of the low-level featural differences that existed between semantic image sets even when scrambled. However, we note that differences in the strength of the signal at the base frequency could reflect other mechanisms than a reduced/inhibited response in younger adults to scrambled stimuli. For instance, differences in the sinusoidal properties of the waveform across conditions could alter the frequency-specific power at the base rate without a change in amplitude of stimulus-related activity. It may be useful for future studies of different age cohorts to include an orthogonal attention task, such as requiring a response to infrequent changes in the colour of the fixation cross^37^. This could indicate whether there are broad attentional differences between cohorts for the two conditions, although it may be possible to diminish processing of the periodic stimulus stream while maintaining attention to the fixation cross. Finally, it is worth highlighting that our report of intact semantic memory in healthy ageing is based on a single dimension, i.e. categorisation of stimuli on the basis of whether they are naturally occurring or man-made. This is one of many possible dimensions on the basis of which objects can be semantically categorised: animacy, pleasantness, colour, size and so on. An integral assessment of semantic memory in ageing may require a more detailed assessment that takes into account the richness of semantic organisation. Similarly, it would be useful for future work to reverse the semantic categories that form standard and oddball images in order to test the stability of the response across different experimental set-ups. Yet, within the limits of our case the basic point remains: automatic semantic categorisation of objects in older adults is preserved. Moreover, by demonstrating this effect using mobile EEG technology with an implicit task of short duration, we present a promising tool for investigation of individual level effects outside of the lab environment.

## Methods

### Participants

This study recruited 29 healthy younger adults between the ages of 20 and 28 (*M* = 21.5 years, *SD* = 1.7) and 21 healthy older individuals between 54 and 84 years old (*M* = 69.9 years, *SD* = 8.8). All participants were right-handed. Healthy young adults were recruited from the University of Bristol student population. The older participants were recruited through a healthy volunteer database at Southmead Hospital, North Bristol NHS Trust, Bristol. Exclusion criteria were a previous diagnosis of dementia, stroke or epilepsy or being currently on medications which may affect the EEG signal. Participation in the experiment was entirely voluntary, did not involve monetary compensation and required informed consent. Ethics approval was granted by the University of Bristol Faculty Ethics Board (approval #10021744261) and the NHS research ethics committee (#16/WS/0153). All procedures were performed in accordance with the NHS ethics guidance (UK Policy Framework for Health & Social Care Research) and informed consent was obtained for all participants.

All older participants took part in a preliminary Montreal Cognitive Assessment (MoCA)^44^. This assessment has a low ceiling and provides a fast assessment of mild cognitive impairment. Of our sample, two healthy older adults scored below the threshold of 26 points and were omitted from the analysis. One older adult’s data was also removed due to noise in the data (see “Data processing and analysis” below). Therefore, our final healthy older adult sample contained 18 participants between 54 and 84 years old (*M* = 70.1, *SD* = 9.0). Two MoCA scores were unavailable, but based on 16 participants, older adults had a mean MoCA score of 28.4 (*SD* = 1.3, range: 26–30; see Supplementary Information for all scores). All older adult volunteers had been previously screened to ensure they did not have a neurological disease.

Given our sample of 47 participants and a 2-way ANOVA with a between-subject factor (Age) and a within-subject factor (Condition), we had 92% power to observe a medium effect of age (f = 0.25). We were interested in a medium effect as previous studies that did find an effect of age reported at least a medium effect^21^.

### Materials

In total, this study used 123 images of natural objects (standard stimuli) and 19 images of man-made objects (oddball stimuli) (for a list of all objects used, see Supplementary Information). The images were taken from the image set created for psycholinguistic research by Moreno-Martínez and Montoro^45^. All images were 540 × 720 pixels and grey scaled for this experiment. The mean luminance of natural images was 0.919 (*SD* = 0.049) and the mean root mean square (RMS) contrast was 0.205 (*SD* = 0.068). The mean luminance of man-made images was 0.877 (*SD* = 0.052) and the mean RMS contrast was 0.246 (*SD* = 0.058). Images were displayed on a white background with each image being displayed for 83.3 ms, with an inter-stimulus interval of 83.3 ms during which a white background was displayed. Images were therefore presented at a rate of 6 Hz, hereafter referred to as the *base frequency*, *F*.

**Figure 4:**
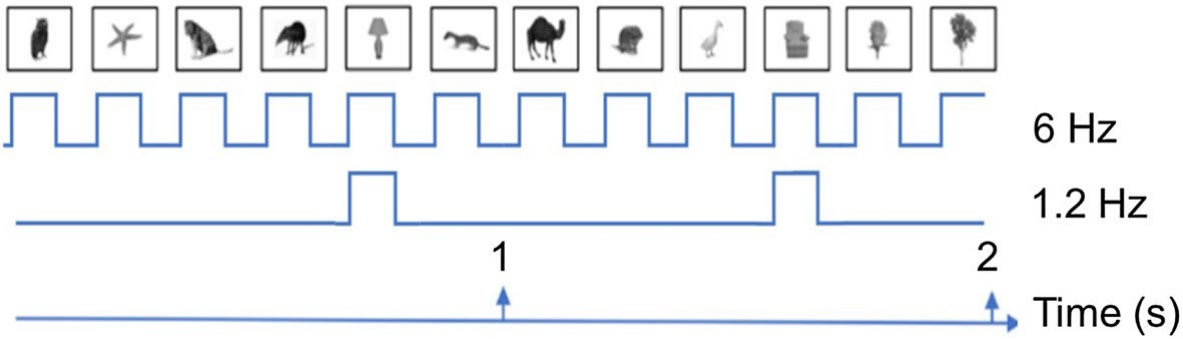
Diagram showing stimulus presentation over two seconds and the hypothesised neural response to the presentation of both standard (middle line) and oddball (bottom) stimuli^45^. Images were taken from the psycholinguistic image set developed and made available by Moreno-Martínez and Montoro.

A full trial lasted 190 s and included 1,140 images displayed at 6 Hz. Within this trial of 1,140 images, every fifth stimulus was an oddball image and the trial can be thought of as 228 consecutive sequences of five stimuli (four standard stimuli; one oddball) (see Fig. 4). The presentation rate for the oddball stimulus (every 5th stimulus) was therefore 1.2 Hz, hereafter referred to as the *oddball frequency*, *f*. For each sequence of five stimuli in the full trial, images were drawn without replacement from the respective standard and oddball image sets. Across a trial, oddball images were presented on average 12 times and standard stimuli 7.4 times.

In addition to the experimental (aka Semantic) condition above, all participants were presented with a control (aka Scrambled) condition in which all images had been scrambled via box scrambling using the Matlab Randblock function (https://uk.mathworks.com/matlabcentral/fileexchange/17981-randblock). In these scrambled images the semantic information on the object was removed whilst the low-level visual content was retained.

An important issue here concerns systematic low-level differences in image properties between natural and man-made categories that might form the basis of observed differences as opposed to semantic categorisation itself. Previous work has suggested that differences in natural and man-made scenes may be detected from such statistical properties^46,47^. This is an inherent problem with making claims of difference between such image types and therefore requires that image type differences are compared to effects using appropriate control stimuli.

We used the GIST descriptor (https://people.csail.mit.edu/torralba/code/spatialenvelope/) developed by Oliva and Torralba^48^ to generate low-level statistical properties of all images used in the experiment. The GIST descriptor creates a 512-point vector for each image that constitutes a high dimensional representation of an image based on spatial frequency and orientation. Images are filtered by a bank of Gabor filters representing eight orientations and four spatial frequencies. This creates 32 filtered images per image which are then summarised across 16 different locations as defined by partitioning the image into a 4 × 4 grid. This produces 16 localized spatial frequency and orientation values for each of the 32 filtered images. In total, 512 values describing the image are generated and represent a low-level description of the image. We generated low-level descriptors for all 142 images (19 natural; 123 man-made) and their scrambled counterparts.

We used a supervised binary classification algorithm implemented by regularized logistic regression (matlab’s lassoglm.m) to determine whether image types (natural vs man-made) of the unscrambled set could be accurately classified on the basis of low-level properties. Using tenfold cross-validation, the algorithm correctly classified 85.7% of stimuli suggesting that image category could be accurately differentiated on the basis of low-level properties. The same procedure was run for scrambled images where classification accuracy was 92.9%. While this suggests that image type could be determined by low-level properties, this is equally true of the control condition. As such, any neural activity related purely to low-level visual differences in the images ought to be evident in both the Semantic and Scrambled condition. In contrast, any condition differences are argued to reflect the disruption of semantic processing caused by scrambling the image and precluding object recognition.

### Procedure

Healthy older adults were tested at the Brain Centre at Southmead Hospital, while the healthy younger adult samples were tested at the University of Bristol. In both locations, testing was conducted in a research environment (e.g. a noise-free testing room) by the same researcher. This same procedure, using the same materials, was followed by both samples. Following written consent, the participant’s demographic information and information relating to exclusion criteria was assessed. The participant then completed the MoCA.

Following the steps above, the EEG headset was positioned on the participant’s head and participants were asked to avoid excessive movement, jaw clenching or blinking during recordings in order to reduce the noise in EEG recordings. In addition to the FPVS task, the EEG session also included a 1-back task^49^ and, for younger participants only, a visual search paradigm task^50^ which are not reported here. At the end of the testing session, all participants took part in a questionnaire evaluating their experience and comfort during the experiment.

Prior to the FPVS task, participants were instructed verbally as well as via written instructions on the screen to passively watch the monitor for the entire paradigm. All participants were presented with the Semantic condition first, followed by the Scrambled condition. The FPVS paradigm was created in Python using the PsychoPy toolbox^51^.

*EEG recording* EEG was recorded using a 14-channel EPOC + headset (Emotiv) with a sample rate of 128 Hz. The channels on the headset (AF3, F7, F3, FC5, T7, P7, O1, O2, P8, T8, FC6, F4, F8, AF4) adhere to the international 10–20 system of electrode locations. Additional reference channels located on the left (in the position similar to P3 channel on the 10–20 system) and right (P4 channel) were used; all other channels were measured relative to the average of the two reference channels. The EEG was recorded using the TestBench software by the same manufacturer.

### Data processing and analysis

EEG data underwent ocular artifact correction procedures in BESA (BESA Gmbh). A 3-min epoch was then taken for both FPVS paradigms which avoided the first 10 s of each recording (to avoid initial ERPs and any eye-movements to stimulation onset). These epochs were linearly detrended, the DC component was removed and then they were subject to correction for any artifacts in excess of ± 200 uV. Artifacts were replaced with zeros and in order to avoid discontinuities in the remaining data we tapered 670 points of data either side of these removed sections using half a Hanning window. For each participant, data from an electrode was not analysed if > 10% of data was removed in this procedure. If half or more electrodes were rejected then the participant was removed from the analysis altogether. For the older cohort, one participant was removed (seven electrodes rejected). The mean number of electrodes removed for the remaining 18 participants was 0.306 (*SD* = 0.668; max 3). For the younger cohort, no participants were removed (number of electrodes removed from 29 participants: *M* = 0.241, *SD* = 0.924; max 6). For analyses using global average data, an individual’s global average was based on all channels remaining after this procedure. For electrode × condition analyses, only participants with data at all channels could be used so these analyses are based on a smaller number of participants as reported in the results.

For each subject and each electrode, amplitude was computed on the 3-min epoch using the Fourier transform. A signal-to-noise ratio (SNR) value was then calculated by dividing the amplitude in each frequency bin by the mean amplitude of surrounding bins within a ± 0.45 Hz range^26,52,53^ excluding the immediately adjacent bins (first neighbouring bin on each side). Given the long epochs, the frequency resolution was 0.0056 Hz and SNR was based on ± 79 bins. For individual analyses, the z-score for each frequency bin was calculated by subtracting the mean and dividing by the standard deviation of surrounding bins (± 0.45 Hz, excluding adjacent bins). The z-score for each frequency bin could then be assessed in relation to levels of alpha (Z = 1.64, *p* = 0.05, one-tailed).

For statistical analysis, SNR was calculated for two values: the base frequency (6 Hz, ***F***) and the mean of the oddball frequency and its harmonics below the base rate (1.2, 2.4, 3.6 and 4.8 Hz, ***f+***).

## Supplementary information

Supplementary Information.
