# Supplementary material for: Fast Periodic Visual Stimulation indexes preserved semantic memory in healthy ageing
Source: Sci Rep. 2020 Aug 4;10:13159. doi: 10.1038/s41598-020-69929-5 (PMC7403314; doi:10.1038/s41598-020-69929-5)
Supplement: Supplementary file 1 — Supplementary Information. [file 41598_2020_69929_MOESM1_ESM.pdf]

Fast Periodic Visual Stimulation indexes preserved semantic memory in healthy ageing

Alex Milton, Alesi Rowland, George Stothart, Phil Clatworthy, Catherine M. Pennington, Nina Kazanina

Supplementary information

Table S1. MoCA scores for Older Adults

Participant	MoCA score
1	29
2	Missing
3	29
4	29
5	29
6	29
7	30
8	29
9	Missing
10	30
11	29
12	27
13	26
14	28
15	29
16	27
17	29
18	26

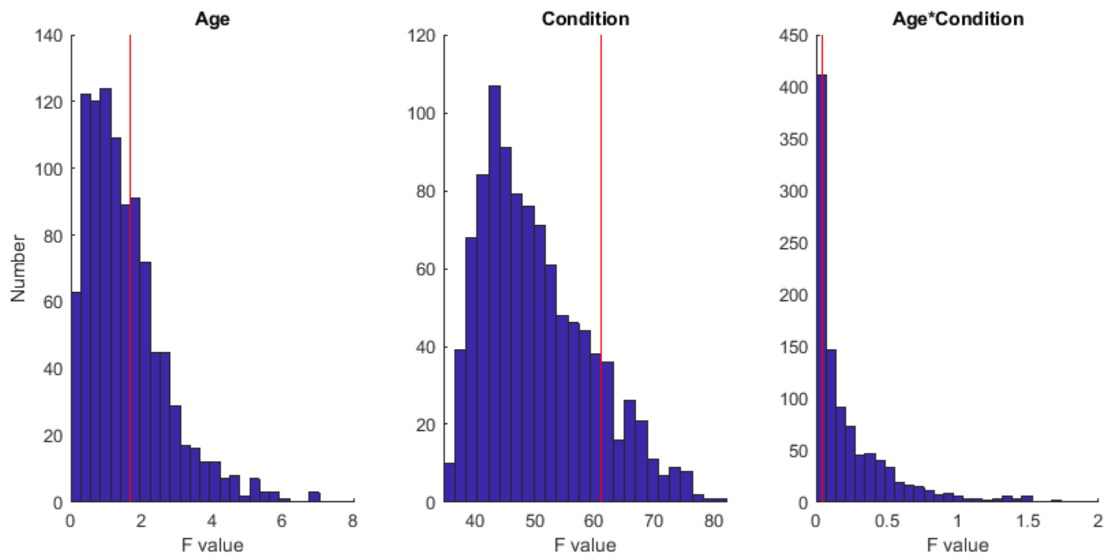

Figure S1. Histograms of *F* values for the subgroup analysis of matched sample sizes reported in the main text. Histograms depict *F* values for the main effect of Age (left panel), Condition (middle panel) and the interaction (right panel). The red line in each plot indicates the values from the original dataset and it can be observed that the original values sit firmly within the observed distribution from analyses of matching group size.

Non-parametric analysis of the topographical effect of Condition

We ran a non-parametric cluster-based permutation test looking at the effect of Condition across the 14 electrodes. Older and Younger adults were pooled into one group. Electrodes considered to be neighbours for the purposes of cluster formation are depicted in Figure S2 below. We used the following connection pattern (indicated by red lines)

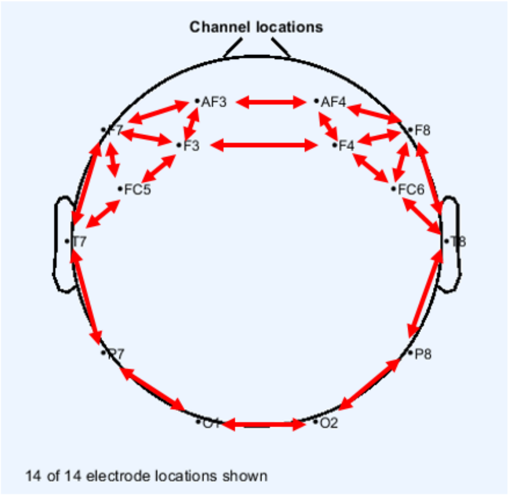

Figure S2. Head map showing electrode placement with red lines indicating connections between neighbouring electrodes used for cluster formation

In order to determine an appropriate non-parametric test statistic, we worked from our wish to test whether the Condition effect (Semantic>Scrambled) holds at each electrode. One test statistic would be to sum the mean Condition difference (Semantic minus Scrambled) for all neighbouring electrodes where Semantic > Scrambled (this latter condition forming an entry threshold where electrodes with Scrambled > Semantic are not considered). As Semantic > Scrambled could be considered the absolute minimal entry threshold for a cluster analysis, we used a more conservative threshold where a larger difference was required. To define this threshold, we averaged the signal across Conditions for each participant at each electrode. We then took the average signal (across Conditions) across all electrodes. This gives us the total average size of the *f*+ response across all electrodes and conditions and therefore biases neither. Our entry threshold for the cluster analysis was only electrodes where Semantic > Scrambled, and this difference was >25% of the total average *f*+ response. This threshold was used to detect electrodes where Condition effects were pronounced.

Using the above parameters and 10,000 permutations in a cluster-based permutation analysis, the observed data revealed a significant effect of Condition (monte carlo *p*=.0001) with a cluster mass including all electrodes but AF3, F7 & F8 (see Figure S3 below)

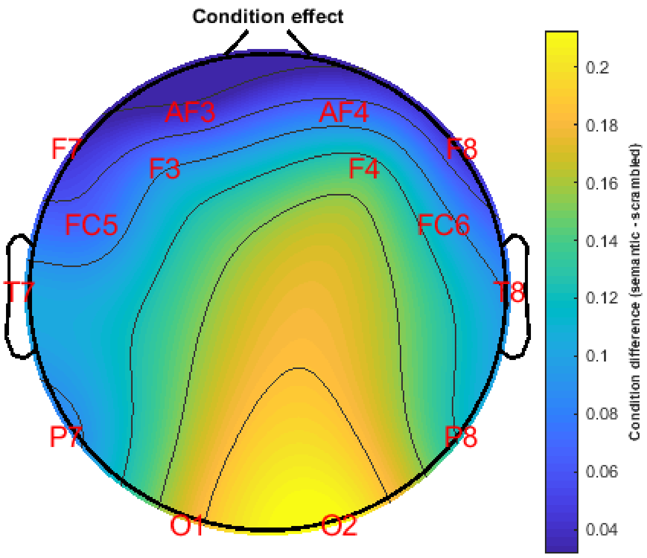

Figure S3. Topographic map showing Condition differences (Semantic *f*+ minus Scrambled *f*+) at each electrode.

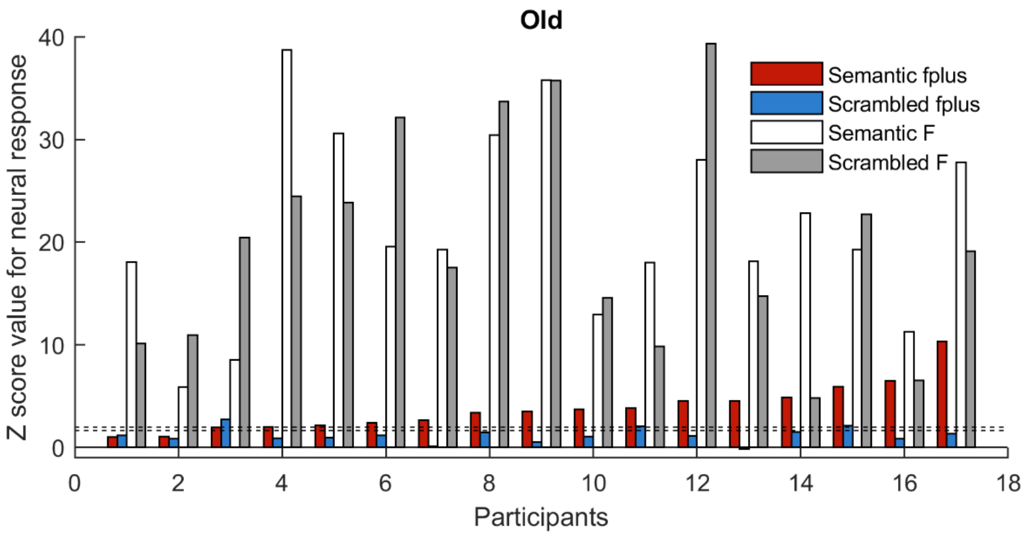

Figure S4. Older adult individual subject *z*-scores for *f*+ (mean of z-scores for all *f*+ frequencies) and *F* for Semantic and Scrambled conditions at electrode O2. Dashed horizontal lines mark z-score threshold for significance at *z*=1.64 (*p*=.05, one-tailed) and *z*=1.96 (*p*=.05, two-tailed).

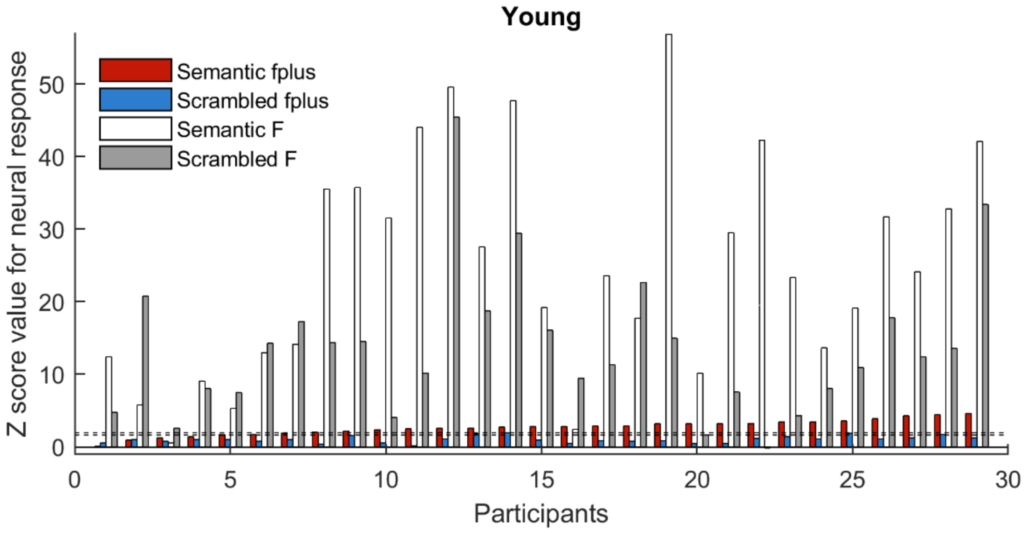

Figure S5. Younger adult individual subject *z*-scores for *f*+ (mean of z-scores for all *f*+ frequencies) and *F* for Semantic and Scrambled conditions at electrode O2. Dashed horizontal lines mark z-score threshold for significance at *z*=1.64 (*p*=.05, one-tailed) and *z*=1.96 (*p*=.05, two-tailed).

Full list of objects used in the experiment

Non-natural objects (19): armchair, bed, castle, cathedral, chair, church, coat, cup, filing cabinet, frying pan, jacket, lamp, mill, saucepan, shanty, shirt, socks, teapot, trousers.

Natural objects (123): araceae, barn owl, bat, bellflowers, bird1, bird2, bird3, blackbird, blackbird1, blackbird2, budgie, budgie2, bustard, carnation, cat, cedar, chaffinch, cheetah, chicken, cockatoo, cow, crocodile, crow, cuckoo, cypress, daisy, dolphin, dromedary, duck, duck1, duck2, eagle, elephant, emu, eucalyptus, fig tree, finch, fir, geranium, gerbil, giraffe, goldfinch, goose, grapes, guinea pig, guinea fowl, gull, hare, hedgehog, hen, hippopotamus, holm oak, horse, hummingbird, killer whale, kingfisher, kiwi, lark, lark2, lark3, lilac, lynx, magpie, manatee, marmot, mole, mouse, olive tree, orchid, ostrich, otter, owl, owl2, palm tree, pansy, parrot, parrot2, parrot3, partridge, pelican, penguin, pheasant, pigeon, pine tree, platypus, poppy, quail, rabbit, rat, raven, rhino, robin, robin2, rooster, rose, sandpiper, seagull, shark, shrew, snake, sparrow, sperm whale, squirrel, squirrel2, starfish, sunflower, swan, tapir, thrush, tiger, tit, tit1, toucan, toucan2, tulip, turtle, vole, vulture, weasel, whale, willow, woodpecker, zebra.
